# Age-Related Differences in the Clinical Profile and Management of Atrial Fibrillation: Results from the Multicentre REGUEIFA Registry

**DOI:** 10.3390/jcm15051955

**Published:** 2026-03-04

**Authors:** Alejandro Manuel López-Pena, Juliana Elices-Teja, Olga Durán-Bobín, Laila González-Melchor, María Vázquez-Caamaño, Emiliano Fernández-Obanza, Eva González-Babarro, Pilar Cabanas-Grandío, Miriam Piñeiro-Portela, Oscar Prada-Delgado, Mario Gutiérrez-Feijoo, Evaristo Freire, Oscar Díaz-Castro, Javier Muñiz, Javier García-Seara, Carlos González-Juanatey

**Affiliations:** 1 Cardiology Department, Hospital Universitario Lucus Augusti, 27003 Lugo, Spain; alejandro.manuel.lopez.pena@sergas.es (A.M.L.-P.); juliana.elices.teja@sergas.es (J.E.-T.); olga.duran.bobin@sergas.es (O.D.-B.); laila.gonzalez.melchor@sergas.es (L.G.-M.); 2 CardioHULA Research Group, Fundación Instituto de Investigación Sanitaria de Santiago de Compostela FIDIS, 27003 Lugo, Spain; 3 Cardiology Department, Hospital San Rafael, 15006 La Coruña, Spain; macrisvc@yahoo.es; 4 Cardiology Department, Hospital Álvaro Cunqueiro and Instituto de Investigación Sanitaria Galicia Sur (IISGS), 36312 Vigo, Spain; emilio.fernandez-obanza-windcheid@sergas.es (E.F.-O.); pilar.cabanas.grandio@sergas.es (P.C.-G.); odiazcastro@hotmail.com (O.D.-C.); 5 Cardiology Department, Hospital Montecelo, 36071 Pontevedra, Spain; eva.gonzalez.babarro@sergas.es; 6 Cardiology Department, Complexo Hospitalario Universitario de A Coruña, Instituto de Investigación Biomédica de A Coruña (INIBIC), 15006 A Coruña, Spain; miriam.pineiro.portela@sergas.es (M.P.-P.); oscar.prada.delgado@sergas.es (O.P.-D.); 7 Cardiology Department, Hospital de Ourense, 32005 Ourense, Spain; mario.gutierrez.feijoo@sergas.es (M.G.-F.); evaristo.freire@sergas.es (E.F.); 8 Grupo de Investigación Cardiovascular, Departamento de Ciencias de la Salud and Instituto de Investigación Biomédica de A Coruña (INIBIC), Universidade da Coruña, CIBERCV, 15006 A Coruña, Spain; javier.muniz.garcia@udc.es; 9 Cardiology Department, Hospital Clínico de Santiago de Compostela, CIBERCV, 15706 A Coruña, Spain; javiergarciaseara@yahoo.es

**Keywords:** atrial fibrillation, elderly patients, anticoagulants, vitamin K antagonists, direct oral anticoagulants, comorbidity, clinical management

## Abstract

**Background/Objectives:** Atrial fibrillation (AF) is the most common sustained arrhythmia in adults, with a prevalence that increases with age. In older patients, its clinical impact is particularly relevant due to higher mortality and greater comorbidity burden. This study aimed to compare patients aged ≥80 years with younger patients in a large AF cohort. **Methods:** The REGUEIFA registry is an observational, prospective, multicentre study including consecutive patients with AF managed by cardiologists. Baseline clinical characteristics, comorbidities, complementary test findings, AF type, therapeutic strategies, anticoagulation patterns, and patient-reported outcomes were compared. **Results:** A total of 1007 patients were included, of whom 18.2% were aged ≥80 years. Older patients showed a higher prevalence of hypertension, renal dysfunction, conduction disorders, chronic obstructive pulmonary disease, and neoplastic disease, along with higher thromboembolic (CHA_2_DS_2_-VASc 3.7 ± 1.04 vs. 2.1 ± 1.49; *p* < 0.001) and haemorrhagic risk (HAS-BLED 1.3 ± 0.8 vs. 0.6 ± 0.7; *p* < 0.001). Permanent AF was more frequent, whereas rhythm control strategies and antiarrhythmic drug use were less common, and quality of life was poorer. Anticoagulation rates were high in both groups (≈90%), with greater use of vitamin K antagonists (VKAs) in older patients, although anticoagulation control was similar. Patients treated with direct-acting oral anticoagulants reported a lower treatment burden and greater perceived benefit than those receiving VKAs. **Conclusions:** Patients aged ≥80 years with AF exhibit greater comorbidity, poorer perceived health status, and higher thromboembolic and haemorrhagic risk. Their management is more often oriented towards rate control strategies and VKA use, while rhythm control approaches are more common in younger patients.

## 1. Introduction

Atrial fibrillation (AF) is the most common sustained cardiac arrhythmia in adults worldwide, with both prevalence and incidence increasing with age [1,2]. In our setting, the estimated prevalence of AF in adults over 40 years of age is 4.4%, reaching up to 17% among those over 80 years [3]. Between 2010 and 2060, the number of people over 55 with AF in the European Union is expected to more than double [4]. A similar trend is projected in the United States, where AF prevalence is anticipated to increase 2.5-fold by 2050, with more than 50% of patients being 80 years or older [2,5].

The importance of AF in older patients extends beyond its epidemiological impact. This arrhythmia is closely associated with increased mortality and a higher burden of age-related comorbidities such as stroke, chronic heart failure, and dementia [2,6,7,8]. This represents a significant burden for patients, public health systems, and healthcare economics [1].

Cardiac thromboembolism attributed to AF accounts for up to one-third of all ischaemic strokes [9]. This proportion increases with age, and a rising trend in the prevalence of AF-related stroke has been reported in recent decades. AF-related strokes are more frequently fatal or disabling and are associated with a higher risk of institutionalisation compared with ischaemic strokes of other aetiologies [9,10,11].

Elderly patients have a higher thromboembolic risk profile (reflected in higher CHA_2_DS_2_-VASc scores) and a higher risk of bleeding (HAS-BLED) [1,12]. Compared with younger patients, they are also more likely to present with renal dysfunction, multiple comorbidities, polypharmacy, and frailty [12,13,14]. These factors not only influence the choice of anticoagulant therapy and clinical management strategies but also limit the available evidence, as elderly patients have traditionally been underrepresented in clinical trials [14,15].

The REgistro GallEgo Intercéntrico de Fibrilación Auricular (REGUEIFA) is an observational, prospective, multicentre registry that includes consecutive patients diagnosed with AF [16]. The aim of this study is to analyse and compare the clinical characteristics and therapeutic strategies used in elderly patients (≥80 years) included in this registry with those of younger patients. This analysis provides novel insights into age-related differences in clinical management, anticoagulation patterns, and patient-reported outcomes, with particular relevance for guiding individualised care in older adults.

## 2. Material and Methods

### 2.1. Study Design and Setting

The REGUEIFA study is a prospective, observational, multicentre registry conducted in a total of eight hospitals in a community health area (Galicia) in north-western Spain. A detailed description of the overall registry design has been previously published [16]; however, the main methodological aspects relevant to the present analysis are summarised below.

A total of 1007 patients with a primary or secondary diagnosis of AF who attended a cardiology clinic or were hospitalised in a cardiology unit between 2 January 2018 and 27 February 2020 were consecutively recruited [16].

### 2.2. Selection of Participants

The study included adult patients, both men and women, aged 18 years or older with a confirmed diagnosis of AF, established by conventional electrocardiogram or by external or implantable Holter monitoring, with documented episodes lasting longer than 30 s. To be eligible, participants needed to have had at least one recorded AF episode in the past year. Patients could be in sinus rhythm at inclusion, provided they met the above diagnostic criteria.

Those who were not considered for, or were not eligible for, long-term follow-up were excluded, as were cases of secondary or transient AF due to reversible causes and patients enrolled in interventional studies that could influence treatment, consultation frequency, or diagnostic procedures. All patients fulfilling these predefined criteria during the study period were consecutively included, and no additional selection or post-enrolment exclusions were performed for the present analysis.

### 2.3. Ethical Statement

The study was conducted in accordance with the principles of the Declaration of Helsinki and was approved by the Clinical Research Ethics Committee (CREC) of Galicia (Spain), reference code 2016/376.

All patients received the patient information sheet and provided written informed consent, in accordance with local regulations, before any study-related procedures were performed.

### 2.4. Data Registry and Quality Assurance and Control

The data were recorded in an electronic data capture form (eCRF) specifically designed for the registry. The information collected was then sent to the coordination centre via secure websites, ensuring confidentiality and compliance with current legislation on data protection and patient autonomy. Each patient was assigned a unique numerical identification code.

A study supervisor reviewed 10% of the eCRFs at participating centres, comparing the data with the original documentation, in accordance with the established quality-control protocol.

### 2.5. Methods and Measurements

For the present analysis, patients were stratified into two age groups: ≥80 years (elderly) and <80 years (reference group).

At the baseline visit, clinical, demographic, and analytical variables were collected, including age and sex, cardiovascular risk factors, results of complementary tests (analytical parameters and echocardiography), characteristics of atrial fibrillation, personal cardiovascular history and comorbidities, antiarrhythmic, cardiovascular and anticoagulant treatments, CHA_2_DS_2_-VASc and HAS-BLED scores, and specific treatment strategies for AF.

Health-related quality of life was assessed using the EuroQol-5D (EQ-5D) questionnaire, which includes five health dimensions (mobility, personal care, everyday activities, pain/discomfort, and anxiety/depression), each with three severity levels. The questionnaire generates a summary index ranging from 0 (worst health status) to 1 (best health status) [17].

Satisfaction with anticoagulant treatment was assessed using the Anti-Clot Treatment Scale (ACTS) questionnaire, which includes two subscales (Burden and Benefit) and two global items [18].

In patients treated with vitamin K antagonists, international normalized ratio (INR) data from the previous 6 months were recorded, and the time in therapeutic range (TTR) was calculated and subsequently analysed according to four age groups.

### 2.6. Statistical Analysis

Quantitative variables were presented as mean ± standard deviation (SD), whereas qualitative variables were presented as percentages.

Differences between the two age groups were analysed using the chi-square test or Fisher’s exact test for qualitative variables, and the non-parametric Mann–Whitney U test (two-sample Wilcoxon rank-sum test) for quantitative variables. The Kruskal–Wallis test was used to compare INR levels over the last 6 months and TTR across age groups.

The results were expressed as *p*-values, with *p* < 0.05 considered statistically significant.

## 3. Results

### 3.1. Basal Characteristics

A total of 1007 patients were included and divided into two age groups: <80 years (*n* = 824, 81.8%) and ≥80 years (*n* = 183, 18.2%). Women represented 32.4% of the cohort, with a higher proportion in the older group (39.9%).

#### Cardiovascular Risk Factors (CVRFs)

Hypertension (HTN) was the most prevalent CVRF (62.3%) and the only one significantly more frequent in patients ≥80 years (74.9%, *p* < 0.001). In contrast, toxic habits (smoking and alcohol consumption) and higher body mass index (BMI) were more common among younger patients (*p* < 0.001). No age differences were observed in the prevalence of diabetes mellitus or dyslipidaemia (Table 1).

### 3.2. Complementary Test

Creatinine levels were significantly higher in the elderly (*p* < 0.001). Left atrial diameter was similar between groups, although left atrial volume was greater in patients aged ≥80 years (*p* = 0.009). Left ventricular ejection fraction remained at the lower limit of normal in both groups (Table 2).

His bundle block-type conduction disturbances were present in 8.9% of patients, with almost a threefold higher prevalence in the elderly (19.7% vs. 6.6%; *p* < 0.001). Right bundle branch block (RBBB) was the most common (56.7%). Both left bundle branch block (LBBB) and RBBB were more frequent in older patients, although without reaching statistical significance (*p* = 0.433 and *p* = 0.862, respectively) (Table 2).

### 3.3. Cardiovascular History

A history of heart failure (HF) was documented in 15%, with no differences between age groups or in NYHA functional class (*p* = 0.277). Valvular heart disease was more common in patients ≥80 years (23.0% vs. 9.1%; *p* < 0.001). No differences were found in the prevalence of cardiomyopathy or coronary artery disease (Table 3).

Overall, 9.9% of patients had an implantable device (implantable cardioverter-defibrillator [ICD] or pacemaker), with a prevalence two to threefold higher among the elderly (24.0% vs. 9.9%; *p* < 0.001). Pacemakers were the most frequent devices overall. By age group, ICDs were more common in younger patients (39.3% vs. 4.6%), while pacemakers predominated in older patients (95.5% vs. 60.7%) (Table 3).

### 3.4. Personal History, Comorbidities, and Previous Thromboembolic Events and Bleeding Episodes

Chronic obstructive pulmonary disease (COPD) was significantly more prevalent in the elderly (20.8% vs. 8.5%; *p* < 0.001), as was neoplastic disease (14.8% vs. 6.8%; *p* < 0.001). No age-related differences were found in the prevalence of thyroid disorders (Table 4).

A total of 6.1% had a history of thromboembolic events, without age differences; cerebrovascular events (ischemic stroke) accounted for nearly half of them (2.9%). Bleeding events occurred in 3.6% and were more frequent in the elderly (6.6% vs. 2.9%; *p* = 0.016) (Table 5).

### 3.5. Risk Scales

**Figure 1:**
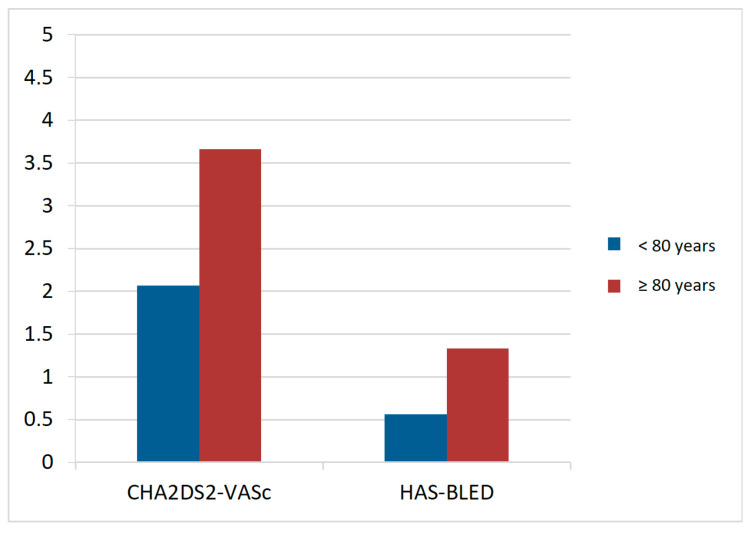
Mean value of the CHA_2_DS_2_-VASc scale and the HAS-BLED scale in patients younger and older than 80 years.

Patients aged ≥80 years had higher thromboembolic risk according to the CHA_2_DS_2_-VASc score (3.7 ± 1.04 vs. 2.1 ± 1.49; *p* < 0.001) and a higher bleeding risk according to the HAS-BLED score (1.3 ± 0.8 vs. 0.6 ± 0.7; *p* < 0.001) (Table 5) (Figure 1).

### 3.6. Characteristics of Atrial Fibrillation

Regarding the clinical type of AF (newly diagnosed, paroxysmal, persistent, long-standing persistent, and permanent AF), significant differences were found between groups (*p* < 0.001). Permanent AF predominated in the older group (54.6%), whereas persistent AF was more frequent in younger patients (30.7%). Paroxysmal AF was also significantly more common among those <80 years (23.9% vs. 14.2%; *p* = 0.004) (Table 6).

Overall, 38.9% of patients had EHRA symptom class IIa, with no differences between age groups (Table 6).

### 3.7. Previous Treatment Strategy (In Non-First Diagnosis of AF)

Rhythm control was applied almost four times more often in younger patients (75.5% vs. 19.8%; *p* < 0.001). Pulmonary vein isolation was also more common in this group (20.1%). Atrioventricular node ablation was performed in three patients (0.7%) (Table 6).

### 3.8. Current Treatment Strategy

Rhythm control remained significantly more common in patients <80 years (72.6% vs. 20.8%; *p* < 0.001). Pacemaker implantation was more frequent in the elderly (9.3% vs. 2.6%; *p* < 0.001), as was atrioventricular node ablation (2.19% vs. 0.6%; *p* = 0.063) (Table 7).

Antiarrhythmic drugs were prescribed more often in younger patients (45.2% vs. 9.8%; *p* < 0.001). Flecainide predominated among younger patients (48.0% vs. 27.8%), while amiodarone (46.4% vs. 55.6%) and sotalol (2.7% vs. 16.7%) were more commonly used in the elderly (Table 8).

β-blockers were also more commonly prescribed in younger patients (73.3% vs. 46.5%; *p* < 0.001). Diuretics were the only therapeutic class more frequently used in the elderly (53.0% vs. 30.2%; *p* < 0.001). No significant age differences were observed in the use of non-dihydropyridine calcium channel blockers, digoxin, ACE inhibitors, angiotensin II receptor blockers (ARBs), statins, antiplatelet agents, or in dual therapy (antiplatelet agents plus anticoagulation) (Table 8).

### 3.9. Anticoagulation

Overall anticoagulation use was high (≈90%), with no age differences (89.1% vs. 92.9%; *p* = 0.123). Vitamin K antagonists (VKA) were the most commonly prescribed agents (58.9%), followed by dabigatran (34.4%), rivaroxaban (28.2%), apixaban (23.9%), and edoxaban (13.4%). Direct oral anticoagulants (DOACs) were more frequently used in younger patients (45.5% vs. 22.4%; *p* < 0.001). Among younger patients, dabigatran was the most common DOAC (37.1%), whereas apixaban predominated in older adults (47.4%) (Table 8).

In the analysis of anticoagulation control (INR and TTR) stratified by four age groups, VKA prescription increased with age (61.5% in ≥80 years vs. 23% in <50 years). However, no age-related differences in INR control were observed during the 6-month follow-up. Mean TTR was 54.9%, with no significant differences among age groups (*p* = 0.740) (Table 9).

### 3.10. EQ-5D and ACTS Questionnaires

The EQ-5D index was significantly lower in patients ≥80 years (0.72 vs. 0.83; *p* < 0.001). Older patients reported more problems with mobility, personal care, daily activities, and pain/discomfort (all *p* < 0.001), with no differences in anxiety/depression (*p* = 0.258) (Table 10).

**Figure 2:**
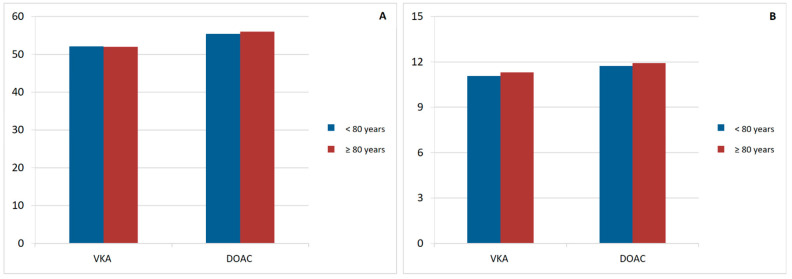
Mean scores of the ACTS Burden (**A**) and Benefit scales (**B**) in anticoagulated octogenarian patients (VKA vs. DOAC), compared with patients younger than 80 years. DOAC: direct-acting oral anticoagulants. VKA: Vitamin K antagonists.

**Figure 3:**
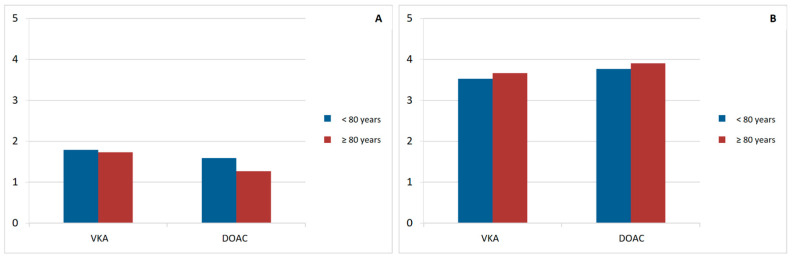
Mean scores of the ACTS General Burden (item 13) (**A**) and General Benefit (item 17) scales (**B**) in anticoagulated octogenarian patients (VKA vs. DOAC), compared with patients younger than 80 years. DOAC: direct-acting oral anticoagulants. VKA: Vitamin K antagonists.

Regarding overall satisfaction with anticoagulation, no age-related differences were observed in ACTS burden, benefit, negative impact, or positive impact scores. However, DOAC-treated patients had higher mean scores (both burden and benefit) than those treated with VKAs, regardless of age (Table 11) (Figure 2 and Figure 3).

## 4. Discussion

The prevalence and incidence of AF increase with age [1]. In this multicentre cohort of 1007 patients with AF, almost one-fifth were aged ≥80 years (18.7%). Age is the main risk factor for its development, with incidence rising up to twofold for every additional 10 years of life [19]. Less than half of the study participants were women (32.4%), which is consistent with the lower incidence of AF in this sex, although this relationship tends to reverse with age, reaching 39.9% in those over 80 years [20]. On average, women develop AF a decade later than men, supporting a possible protective hormonal effect and making the onset of arrhythmia less likely before menopause [20,21].

Older patients more frequently present with multimorbidity and frailty, conditions closely linked to worse clinical outcomes [1]. However, the elderly population is highly heterogeneous, and clinical vulnerability may be partly reflected in patient-reported outcomes. In this context, older patients in our cohort showed poorer functional status in several EQ-5D domains (mobility, personal care, and daily activities), suggesting greater dependency and frailty-related characteristics. Epidemiological evidence indicates that the concomitant presence of multiple comorbidities increases the risk of developing AF, and, in turn, patients with AF have a high burden of cardiovascular and non-cardiovascular comorbidity, as well as high rates of multimorbidity [1,22].

In our cohort, hypertension was the most prevalent cardiovascular risk factor and the only one significantly more frequent in individuals aged ≥80 years. This finding is consistent with studies identifying hypertension as one of the main determinants of AF, given that chronically elevated blood pressure promotes left atrial enlargement, wall stress, and fibrotic remodelling [19,23]. In contrast, toxic habits (smoking and alcohol consumption) and higher BMI were more common in younger patients. In this group, AF is often related to reversible triggers such as hyperthyroidism, alcohol consumption, or intense exercise, usually in structurally normal hearts; whereas in older adults, the arrhythmia more often reflects prolonged cardiovascular damage, chronic inflammation, and atrial fibrosis [24].

The higher creatinine levels observed in older people reflect the progressive decline in renal function associated with ageing. Chronic kidney disease has a bidirectional relationship with AF: renal dysfunction predisposes to its onset through volume overload and electrolyte imbalances, while AF can contribute to renal deterioration through haemodynamic, inflammatory, and thromboembolic mechanisms, as well as by modifying the safety profile of anticoagulation [24,25]. In recent years, sodium–glucose cotransporter 2 (SGLT2) inhibitors have emerged as disease-modifying therapies in patients with heart failure and chronic kidney disease. Beyond their established cardio-renal benefits, increasing evidence suggests a potential role in AF prevention. A recent meta-analysis of randomized controlled trials reported a significant reduction in incident AF among patients treated with SGLT2 inhibitors, particularly in those with heart failure and reduced ejection fraction. These findings may be especially relevant in elderly populations, in whom heart failure, renal dysfunction, and multimorbidity are highly prevalent [26].

From a structural standpoint, left atrial enlargement is associated with an increased risk of developing AF [27]. In our cohort, elderly patients had larger atrial sizes, which may help explain the higher prevalence of AF in older age groups.

Valvular disease, COPD, and neoplastic disease were also significantly more frequent in patients ≥80 years. These findings are consistent with recent studies describing a bidirectional relationship between these comorbidities and AF in the elderly, associated with greater clinical frailty, susceptibility to arrhythmia, and worse clinical outcomes [28,29].

Conduction disorders, particularly bundle branch blocks, were three times more frequent in patients aged ≥80 years, in line with studies documenting progressive structural and electrical remodelling of the conduction system with age, favoured by fibrosis and degeneration. The predominance of right bundle branch block in our population is consistent with findings reported in previous geriatric cohorts [5]. In parallel, we found a higher prevalence of implanted cardiac devices in older patients, mainly pacemakers, a pattern documented in contemporary registries showing that the need for cardiac pacing increases significantly with age, while implantable cardioverter-defibrillators are preferentially used in younger patients with lower comorbidity burden [5].

In addition to its association with increased cardiovascular risk and mortality, AF has a negative impact on quality of life. In our study, 38.9% of patients had EHRA class IIa symptoms, with no differences between groups, although older patients showed poorer perceived health in the EQ-5D questionnaire. Atypical presentations are common in elderly populations, with palpitations present in only 1 in 10 patients ≥80 years [5]. Thirty-three per cent of elderly patients were asymptomatic (EHRA I). This pattern aligns with screening studies in individuals aged 75–76, where AF detection increased by 33%, highlighting the high proportion of asymptomatic AF in older age groups [30].

Permanent AF was the most common form of presentation among the elderly, whereas persistent and paroxysmal AF predominated in younger patients. This pattern is consistent with other registries describing a progressive increase in non-self-limiting forms of AF (persistent and permanent) with advancing age [31]. Furthermore, sustained forms of AF have been associated with a higher risk of cardiovascular death and hospitalisation for heart failure compared with paroxysmal AF [32].

The rhythm-control strategy was used almost four times less frequently in older patients, aligning with current evidence. The benefit of early rhythm control progressively diminishes with age: although it significantly reduces cardiovascular events in patients <75 years, this effect is attenuated in those ≥75 years, where it loses statistical significance [33]. This reduced efficacy may be related to more advanced structural and electrical atrial remodelling and a higher burden of comorbidities, which limit therapeutic options [5].

Fewer antiarrhythmic drugs (AADs) were prescribed in the elderly, with amiodarone and sotalol predominating, whereas flecainide was more frequent in younger patients. The management of AADs in older patients is complex due to the risk of proarrhythmia, frailty, and drug interactions. Amiodarone is used more frequently because of its efficacy, its utility in patients with ventricular dysfunction, and its lack of need for dose adjustment in renal or hepatic impairment, although its long-term toxicity remains a limitation. Fewer beta-blockers were also used in older patients, which is relevant given the higher prevalence of sinus node dysfunction and AV block, although recommended target rates do not differ from those of younger patients [5].

Pulmonary vein ablation was more common in younger patients. Although studies in elderly populations show comparable success rates and no significant increase in complications, these patients more frequently require repeat procedures, remain more dependent on AADs, and present with a more complex atrial substrate due to age-related fibrosis [34]. In contrast, more definitive strategies such as atrioventricular node ablation with pacemaker implantation are usually reserved for elderly patients with permanent AF, refractoriness to drug treatment, or significant frailty, offering effective rate control when pulmonary vein isolation is not an appropriate option [5].

Our registry included a large sample of elderly anticoagulated patients with AF (92.9%). These patients had significantly higher thromboembolic (CHA_2_DS_2_-VASc 3.7 ± 1.04 vs. 2.1 ± 1.49) and haemorrhagic (HAS-BLED 1.3 ± 0.8 vs. 0.6 ± 0.7) risks than younger individuals. Previous studies have also reported that elderly patients tend to have greater cognitive impairment, dependence, and frailty, along with a higher number of comorbidities and elevated thromboembolic and haemorrhagic risk [5,35]. However, none of these variables should be considered in isolation as absolute contraindications to anticoagulation. This is particularly relevant given that anticoagulation discontinuation in older patients with AF is common and is associated with increased mortality and adverse events [35].

In our cohort, most patients were anticoagulated with VKAs, and the quality of VKA anticoagulation was not associated with age. The mean TTR was 54.9%, with no statistically significant differences between age groups, and INR control was similar. This is of interest, since more than 5% of octogenarians have severe renal disease, a condition in which DOACs are not recommended [36]. Similarly, Costa et al. observed that elderly patients achieved comparable anticoagulation quality with VKAs despite having higher levels of comorbidity and polypharmacy [37]. Likewise, in the Swedish AuricuLA registry, older patients managed warfarin therapy as well as or even better than younger patients [38].

DOACs have demonstrated a more favourable benefit–risk profile than VKAs in elderly patients, both in clinical trials and in real-world practice [1,5]. In this context, the mean burden-benefit score was higher among those receiving DOACs, regardless of age, suggesting a greater perception of usefulness and convenience with these drugs. Although DOACs may be preferable in many older patients due to more predictable pharmacokinetics, fewer interactions, and the absence of routine laboratory monitoring, evidence shows that anticoagulation can be managed safely and effectively with both DOACs and VKAs [5]. Thus, frail, polymedicated patients aged ≥75 years who are clinically stable on VKA therapy may continue on this regimen without requiring a switch to a DOAC [39].

## 5. Conclusions

In this large cohort of patients with AF, those aged ≥80 years had a more unfavourable clinical profile, with greater comorbidity, higher thromboembolic and haemorrhagic risk, and poorer quality of life than younger patients. Permanent AF and rate-control strategies were predominant, whereas rhythm control and the use of antiarrhythmic drugs were less common. Anticoagulation rates were high across all groups, with greater use of VKAs in the elderly, although INR control was similar. VKAs were associated with a greater perceived burden and negative impact compared with DOACs. These findings highlight the distinct clinical characteristics and management patterns observed in elderly patients with AF in contemporary practice, and suggest that individualized therapeutic decisions balancing efficacy, safety, and quality of life may be particularly relevant in this population.

## 6. Study Limitations

This is an observational real life study; however, it included a large number of patients from different regions, which strengthens the external validity of the results. Given its descriptive design, findings should be interpreted as reflecting real-world patterns of care rather than establishing causal relationships, providing comprehensive information on clinical characteristics, therapeutic strategies, quality of life, and patient satisfaction with treatment in a broad and diverse AF population.

This registry includes patients managed by cardiologists in a multicentre setting, which may involve closer follow-up and potentially higher treatment adherence compared with patients managed in other settings. However, these findings are consistent with previous evidence suggesting that anticoagulation control is not strongly influenced by age, while providing novel insights into age-related differences in contemporary clinical practice.

Given the observational design of the study, differences in patient-reported treatment burden and benefit may reflect both pharmacological factors and baseline clinical differences between patients. However, these findings are plausible and consistent with previous literature showing a more favourable benefit–risk profile and higher patient satisfaction associated with DOACs compared with VKAs.

## Figures and Tables

**Table 1:** Basal characteristics. Data are expressed as absolute numbers (*n*) and percentages, or as mean ± standard deviation.

Variables	Whole Cohort		Age < 80 Years		Age ≥ 80 Years		*p* Value
	*n*	%	*n*	%	*n*	%	
Sex (female)	326	32.37	253	30.70	73	39.89	0.016
Body mass index (BMI)	1004	29.62 ± 5.02	821	29.96 ± 5.15	183	28.12 ± 4.06	<0.001
Tobacco							<0.001
Never	658	65.34	521	63.23	137	74.86	
Current smoker	86	8.54	84	10.19	2	1.09	
Recent ex-smoker (<6 months)	19	1.89	17	2.06	2	1.09	
Former smoker (≥6 months)	244	24.23	202	24.51	42	22.95	
Smokes (yes)	86	8.54	84	10.19	2	1.09	<0.001
Alcohol							<0.001
No	419	41.61	319	38.71	100	54.64	
Light	495	49.16	416	50.49	79	43.17	
Moderate	76	7.55	73	8.86	3	1.64	
High	17	1.69	16	1.94	1	0.55	
Drink alcohol (yes)	588	58.39	505	61.29	83	45.36	<0.001
Hypertension	627	62.26	490	59.47	137	74.86	<0.001
Diabetes mellitus							0.217
No	818	81.23	677	82.16	141	77.05	
Type 1	6	0.60	5	0.61	1	0.55	
Type 2	183	18.17	142	17.23	41	22.40	
Diabetes mellitus (yes)	189	18.77	147	17.84	42	22.95	0.109
Dyslipidemia	487	48.36	398	48.30	89	48.63	0.935

**Table 2:** Complementary tests. LVEF: left ventricular ejection fraction; LA: left atrium; LBBB: left bundle block; RBBB: right bundle block. Data are expressed as absolute numbers (*n*) and percentages, or as mean ± standard deviation.

Variables	Whole Cohort		Age < 80 Years		Age ≥ 80 Years		*p* Value
	*n*	%	*n*	%	*n*	%	
Creatinine (mg/dL)	956	0.99 ± 0.36	780	0.97 ± 0.35	176	1.07 ± 0.39	<0.001
LVEF (%)	667	56.06 ± 12.73	543	55.87 ± 12.85	124	56.91 ± 12.18	0.321
Diameter of LA (mm)	524	43.96 ± 11.7	426	44.13 ± 11.46	98	43.22 ± 12.76	0.768
Volume of LA (mL/m^2^)	205	56.73 ± 32.94	165	54.56 ± 32.09	40	65.65 ± 35.25	0.009
Bundle block	90	8.94	54	6.55	36	19.67	<0.001
LBBB	38/90	42.22	21/54	38.89	17/36	47.22	0.433
RBBB	51/90	56.67	31/54	57.41	20/36	55.56	0.862

**Table 3:** Cardiovascular history. ICD: implantable cardioverter; HF: heart failure; NYHA: New York Heart Association. Data are expressed as absolute numbers (*n*) and percentages.

Variables	Whole Cohort		Age < 80 Years		Age ≥ 80 Years		*p* Value
	*n*	%	*n*	%	*n*	%	
Heart failure	150	14.90	118	14.32	32	17.49	0.277
HF based on NYHA functional class	150		118		32		0.277
I	27	18.00	24	20.34	3	9.38	
II	82	54.67	65	55.08	17	53.13	
III	40	26.67	28	23.73	12	37.50	
IV	1	0.67	1	0.85	0	0.00	
NYHA functional class ≥ II	123/150	82.00	94/118	79.66	29/32	90.63	0.198
Coronary disease	116	11.52	91	11.04	25	13.66	0.316
Valve disease	117	11.62	75	9.10	42	22.95	<0.001
Cardiomyopathy	78	7.75	64	7.77	14	7.65	0.957
Previous device	100	9.93	56	6.80	44	24.04	<0.001
Type of device	100		56		44		<0.001
Pacemaker carrier	76	76.00	34	60.71	42	95.45	
ICD carrier	24	24.00	22	39.29	2	4.55	

**Table 4:** Concomitant diseases. COPD: chronic obstructive pulmonary disease. Data are expressed as absolute numbers (*n*) and percentages.

Variables	Whole Cohort		Age < 80 Years		Age ≥ 80 Years		*p* Value
	*n*	%	*n*	%	*n*	%	
COPD	108	10.72	70	8.50	38	20.77	<0.001
Neoplasms	83	8.24	56	6.80	27	14.75	<0.001
Hyperthyroidism	19	1.89	15	1.82	4	2.19	0.763
Hypothyroidism	66	6.55	53	6.43	13	7.10	0.740

**Table 5:** Thromboembolic and bleeding events and risk scores. Data are expressed as absolute numbers (*n*) and percentages, or as mean ± standard deviation.

Variables	Whole Cohort		Age < 80 Years		Age ≥ 80 Years		*p* Value
	*n*	%	*n*	%	*n*	%	
Thromboembolic events	61	6.06	47	5.70	14	7.65	0.318
Ischemic stroke (cerebrovascular event)	29	2.88	22	2.67	7	3.83	0.461
Hemorrhagic events	36	3.57	24	2.91	12	6.56	0.016
CHA_2_DS_2_-VASc scale	1007	2.35 ± 1.54	824	2.07 ± 1.49	183	3.66 ± 1.04	<0.001
HAS-BLED scale	1007	0.7 ± 0.78	824	0.56 ± 0.71	183	1.33 ± 0.8	<0.001

**Table 6:** Characteristics of previous atrial fibrillation management strategies. AF: atrial fibrillation; AV: atrioventricular; EHRA: European Heart Rhythm Association. Data are expressed as absolute numbers (*n*) and percentages.

Variables	Whole Cohort		Age < 80 Years		Age ≥ 80 Years		*p* Value
	*n*	%	*n*	%	*n*	%	
EHRA classification							0.197
I	340	33.76	280	33.98	60	32.79	
IIa	392	38.93	330	40.05	62	33.88	
IIb	173	17.18	131	15.90	42	22.95	
III	95	9.43	77	9.34	18	9.84	
IV	7	0.70	6	0.73	1	0.55	
Clinical type of AF							<0.001
First diagnosis	231	22.94	189	22.94	42	22.95	
Paroxysmal	223	22.14	197	23.91	26	14.21	
Persistent	265	26.32	253	30.70	12	6.56	
Long duration persistent	26	2.58	23	2.79	3	1.64	
Permanent	262	26.02	162	19.66	100	54.64	
Paroxysmal AF	223	22.14	197	23.91	26	14.21	0.004
Previous strategy (if not first diagnosis of AF)							
Rhythm control	403/627	64.27	378/501	75.45	25/126	19.84	<0.001
Ablation of AF	403		378		25		0.021
No	324	80.40	299	79.10	25	100.00	
Isolation of pulmonary veins	76	18.86	76	20.11	0	0.00	
AV node ablation	3	0.74	3	0.79	0	0.00	

**Table 7:** Current clinical management strategies. AV: atrioventricular. Data are expressed as absolute numbers (*n*) and percentages.

Variables	Whole Cohort		Age < 80 Years		Age ≥ 80 Years		*p* Value
	*n*	%	*n*	%	*n*	%	
Rhythm control strategy	636	63.16	598	72.57	38	20.77	<0.001
Implantation of devices	38	3.77	21	2.55	17	9.29	<0.001
Rate control interventions AV node ablation	9	0.89	5	0.61	4	2.19	0.063

**Table 8:** Pharmacological treatment. ACEIs: angiotensin II converting enzyme inhibitors; ARAs: angiotensin II receptor antagonists; DOACs: direct-acting oral anticoagulants. Data are expressed as absolute numbers (*n*) and percentages.

Variables	Whole Cohort		Age < 80 Years		Age ≥ 80 Years		*p* Value
	*n*	%	*n*	%	*n*	%	
Antiarrhythmic treatment	390	38.73	372	45.15	18	9.84	<0.001
Group I and/or III antiarrhythmic drugs	389/390	99.74	371/372	99.73	18/18	100.00	1.000
Type of group I and/or III antiarrhythmic drugs	389		371		18		0.051
Amiodarone	182	46.79	172	46.36	10	55.56	
Dronedarone	6	1.54	6	1.62	0	0.00	
Sotalol	13	3.34	10	2.70	3	16.67	
Flecainide	183	47.04	178	47.98	5	27.78	
Propafenone	5	1.29	5	1.35	0	0.00	
Beta-blockers	689	68.42	604	73.30	85	46.45	<0.001
Non-dihydropyridinic calcium antagonists	54	5.36	48	5.83	6	3.28	0.205
Digoxin	62	6.16	51	6.19	11	6.01	0.928
ACEIs/ARAs	517	51.34	427	51.82	90	49.18	0.518
Diuretics	346	34.36	249	30.22	97	53.01	<0.001
Statins	466	46.28	386	46.84	80	43.72	0.443
Antiplatelet medication	43	4.27	33	4.00	10	5.46	0.377
Anticoagulation + Antiplatelet drugs	27	2.68	22	2.67	5	2.73	1.000
Anticoagulation	904	89.77	734	89.08	170	92.90	0.123
Type of anticoagulant	904		734		170		<0.001
Vitamin K antagonist	532	58.85	400	54.50	132	77.65	
DOACs	372	41.15	334	45.50	38	22.35	
Type of DOACs	372		334		38		<0.001
Dabigatran	128	34.41	124	37.13	4	10.53	
Edoxaban	50	13.44	41	12.28	9	23.68	
Rivaroxaban	105	28.23	98	29.34	7	18.42	
Apixaban	89	23.92	71	21.26	18	47.37	

**Table 9:** ACTS scale. Comparison of INR levels in the last 6 months of patients receiving vitamin K antagonists (VKAs) between age groups. INR: international normalized ratio, TTR: time in the therapeutic range. Data are expressed as absolute numbers (*n*) and percentages, or as mean ± standard deviation.

Variable	Whole Cohort (*n* = 1007)	Age < 50 Years (*n* = 70)	Age 50–64 Years (*n* = 312)	Age 65–79 Years (*n* = 442)	Age ≥ 80 Years (*n* = 183)	*p* Value
On vitamin K antagonist therapy (*n*)	468/1000	16/70	92/308	248/440	112/182	
On vitamin K antagonist therapy (%)	46.80	22.86	29.87	56.36	61.54	<0.001
INR month 1 (*n*)	438	14	86	235	103	
INR month 1, mean ± SD	2.46 ± 0.7	2.14 ± 0.58	2.51 ± 0.66	2.47 ± 0.72	2.43 ± 0.69	0.284
INR month 2 (*n*)	434	14	86	234	100	
INR month 2, mean ± SD	2.53 ± 0.82	2.24 ± 0.64	2.59 ± 0.88	2.53 ± 0.83	2.5 ± 0.79	0.522
INR month 3 (*n*)	428	14	84	231	99	
INR month 3, mean ± SD	2.57 ± 0.92	2.66 ± 1.31	2.55 ± 0.79	2.56 ± 0.9	2.61 ± 1.02	0.937
INR month 4 (*n*)	418	14	83	223	98	
INR month 4, mean ± SD	2.56 ± 1	2.44 ± 1.19	2.62 ± 0.97	2.52 ± 1.01	2.62 ± 0.98	0.522
INR month 5 (*n*)	414	14	82	221	97	
INR month 5, mean ± SD	2.57 ± 0.99	2.35 ± 0.95	2.52 ± 0.87	2.6 ± 0.98	2.58 ± 1.1	0.715
INR month 6 (*n*)	393	12	81	208	92	
INR month 6, mean ± SD	2.4 ± 0.94	2.15 ± 0.68	2.42 ± 1.31	2.42 ± 0.88	2.37 ± 0.7	0.668
TTR (*n*)	434	14	86	234	100	
TTR, mean ± SD	54.88 ± 26.27	47.35 ± 30.72	55.39 ± 25.47	54.65 ± 25.94	56.04 ± 27.29	0.740

**Table 10:** EQ-5D questionnaire. Data are expressed as absolute numbers (*n*) and percentages, or as mean ± standard deviation. Statistically significant *p*-values are presented in the table.

Variables	Whole Cohort		Age < 80 Years		Age ≥ 80 Years		*p* Value
	*n*	%	*n*	%	*n*	%	
EQ-5D questionnaire	941	93.45	786	95.39	155	84.70	<0.001
Mobility	941		786		155		<0.001
I have no problems walking	648	68.86	583	74.17	65	41.94	
I have some problems walking	288	30.61	200	25.45	88	56.77	
I have to stay in bed	5	0.53	3	0.38	2	1.29	
Personal care	941		786		155		0.001
I have no problems with personal care	862	91.60	732	93.13	130	83.87	
I have some problems washing or dressing	73	7.76	51	6.49	22	14.19	
I am unable to wash or dress myself	6	0.64	3	0.38	3	1.94	
Daily life activities	941		786		155		<0.001
I have no problems performing daily life activities	722	76.73	625	79.52	97	62.58	
I have some problems performing daily life activities	202	21.47	152	19.34	50	32.26	
I am unable to perform daily life activities	17	1.81	9	1.15	8	5.16	
Pain/discomfort	941		786		155		<0.001
I have no pain or discomfort	652	69.29	567	72.14	85	54.84	
I have moderate pain or discomfort	262	27.84	196	24.94	66	42.58	
I have much pain or discomfort	27	2.87	23	2.93	4	2.58	
Anxiety/depression	941		786		155		0.258
I am not anxious or depressed	655	69.61	554	70.48	101	65.16	
I am moderately anxious or depressed	254	26.99	204	25.95	50	32.26	
I am very anxious or depressed	32	3.40	28	3.56	4	2.58	
EQ-5D index (summarizing score)	941	0.82 ± 0.21	786	0.83 ± 0.2	155	0.72 ± 0.21	<0.001

**Table 11:** ACTS scale. Data are expressed as absolute numbers (*n*) and mean ± standard deviation. Statistically significant *p*-values are presented in the table.

Variables	Whole Cohort		Age < 80 Years		Age ≥ 80 Years		*p* Value
	*n*	%	*n*	%	*n*	%	
Burden scale	738	53.43 ± 7.95	609	53.54 ± 7.95	129	52.92 ± 7.96	0.241
Benefit scale	738	11.34 ± 2.55	609	11.31 ± 2.67	129	11.45 ± 1.94	0.912
General burden scale	738	1.69 ± 1.01	609	1.71 ± 1.02	129	1.62 ± 0.98	0.267
General benefit scale	738	3.63 ± 1.01	609	3.62 ± 1.04	129	3.69 ± 0.87	0.684

## Data Availability

Data will be made available on reasonable request to the corresponding author.
